# Supplementary material for: Molecular-Simulation–Inspired Synthesis of [6]-Prismane via Photoisomerisation of Octafluoro[2.2]paracyclophane
Source: Molecules. 2024 Feb 8;29(4):783. doi: 10.3390/molecules29040783 (PMC10891812; doi:10.3390/molecules29040783)
Supplement: Supplementary file 1 [file molecules-29-00783-s001.zip › molecules-2770423-supplementary.pdf]

Supplementary Information

Molecular Simulation–Inspired Synthesis of [6]-Prismane via the Photoisomerisation of Octafluoro[2.2]paracyclophane

Yoichi Hosokawa[1]*, Shuji Kajiya[1], Ayako Ohshima[1], Satoshi Kawata[2], Nobuhiro Ishida[1], and Arimitsu Usuki[1]

[1]*Toyota Central R&D Labs., Inc., 41-1, Yokomichi, Nagakute, Aichi 480-1192, Japan*

[2]*Department of Chemistry, Faculty of Science, Fukuoka University, 19-1 Nanakuma 8-Chome, Jonan-ku, Fukuoka 814-0180, Japan*

Contents

Figure S1. Calculation flow chart.

Figure S2. Results of molecular mechanics calculations performed for [6]-prismane derivatives.

Figure S3. Heat of Formation for selected prismane derivatives and their precursors.

Figure S4. C···C distances in **11** optimised by MOPAC PM7.

Figure S5. Highest occupied molecular orbital (HOMO)–LUMO gaps of selected cyclophanes.

Figure S6. ^{1}H NMR spectra (400 MHz, $CD_3CN/D_2O/(CD_3)_2SO$ = 2/1/8, v/v/v) of **10** recorded after (**a**) 15 and (**b**) 45 min irradiation.

Figure S7. Ultraviolet (UV)–Vis spectra of selected reaction systems.

Figure S8. ^{19}F NMR spectra (373 MHz) of **10** photoirradiated in different solvents.

Figure S9. NMR spectra (400 MHz) of **10** in (**a**) CD_3CN/D_2O/DMSO-d_6 and (**b**) D_2O/DMSO-d_6.

Figure S10. ^{19}F NMR spectra (373 MHz) of the photoreaction mixture recorded before and after heating.

Figure S11. ^{13}C NMR spectra (100 MHz) of (**a**) cyclophane **10**, (**b**) reaction solution after UV irradiation, and (**c**) evaporated reaction solution.

Figure S12. ^{19}F NMR spectra (373 MHz) of the reaction solution kept under vacuum for (**a**) 0, (**b**) 2, and (**c**) 10 min.

Figure S13. NMR spectra of cyclophane **10** assigned by ROYAL probe HFX at 25 °C.

Figure S14. NMR spectra of **11** by ROYAL probe HFX at 25 °C.

Figure S15. ORTEP drawing of **10** (50% probability).

Figure S16. (**a**) Single-molecule and (**b**) packing (*a*-axis) structures of **10**.

Figure S17. Infrared spectra of **10** and the recovered precipitate.

Figure S18. ^{19}F NMR (373 MHz) spectrum of the photoreaction solution with a $CFCl_3$ standard.

Table S1. Cartesian coordinates of the PM7 optimized geometry of norbornadiene **12** (in Å).

Table S2. Cartesian coordinates of the PM7 optimized geometry of quadricyclane **13** (in Å).

Table S3. Cartesian coordinates of the PM7 optimized geometry of fluorinated norbornadiene **14** (in Å).

Table S4. Cartesian coordinates of the PM7 optimized geometry of fluorinated quadricyclane **15** (in Å).

Table S5. Cartesian coordinates of the PM7 optimized geometry of cyclophane **10** (in Å).

Table S6. Cartesian coordinates of the PM7 optimized geometry of prismane **11** (in Å).

Table S7. Cartesian coordinates of the PM7 optimized geometry of cyclophane **17** (in Å).

Table S8. Cartesian coordinates of the PM7 optimized geometry of cyclophane **16** (in Å).

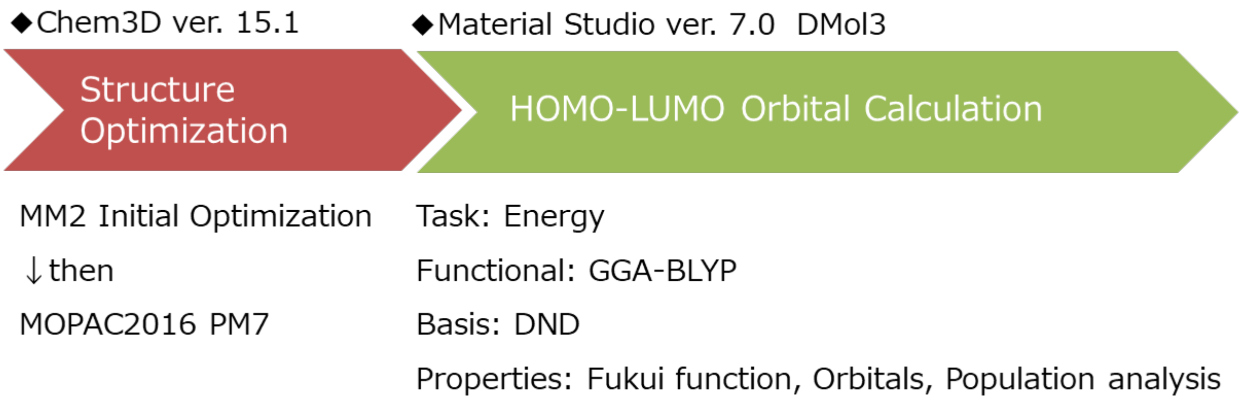

Figure S1. Calculation flow chart.

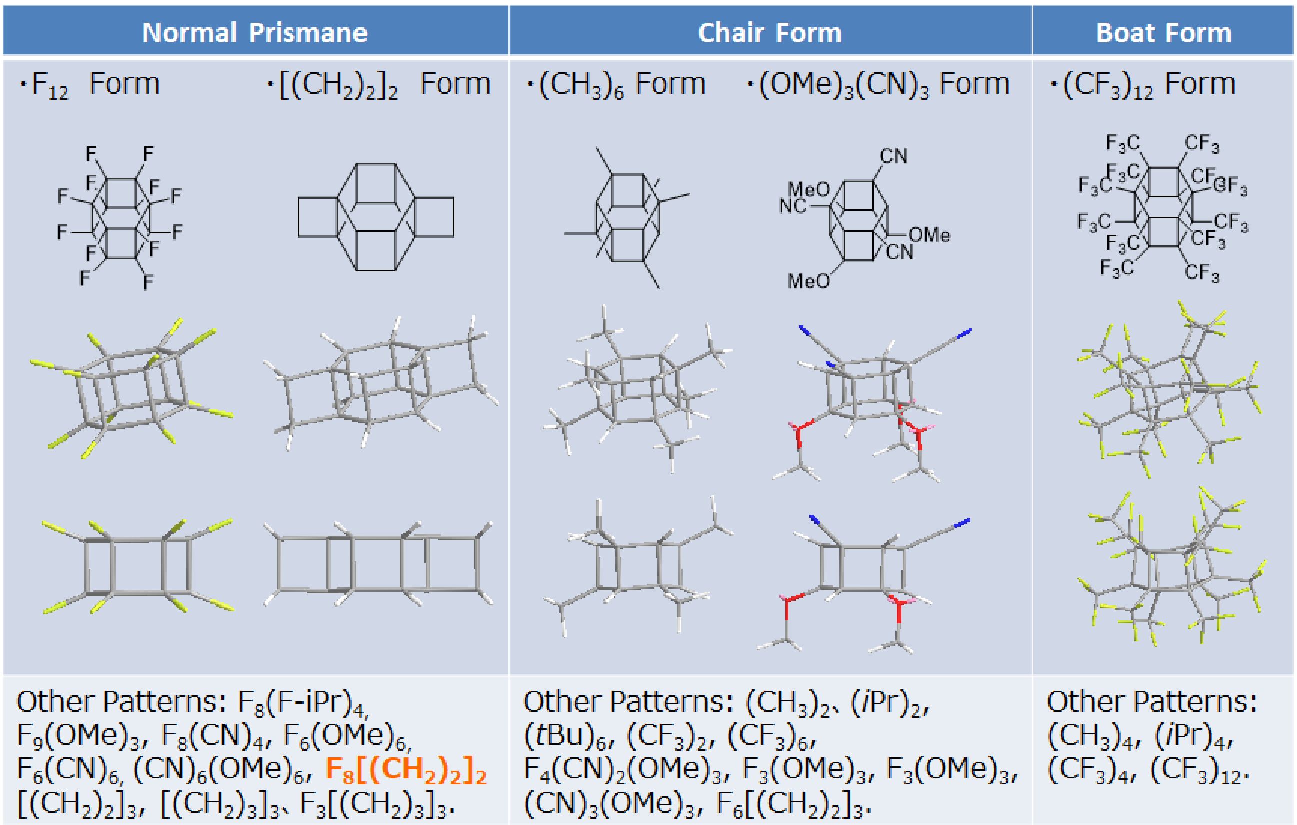

Figure S2. Results of molecular mechanics calculations performed for [6]-prismane derivatives.

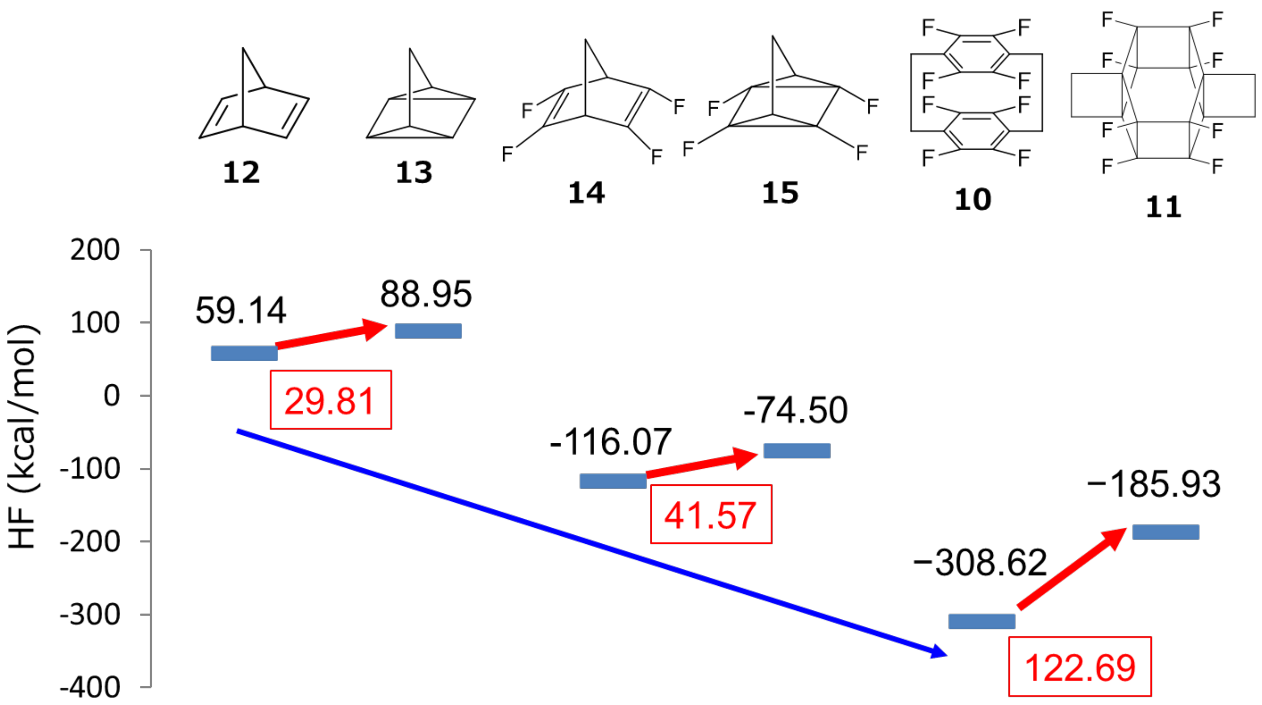

Figure S3. Heat of Formation for selected prismane derivatives and their precursors.

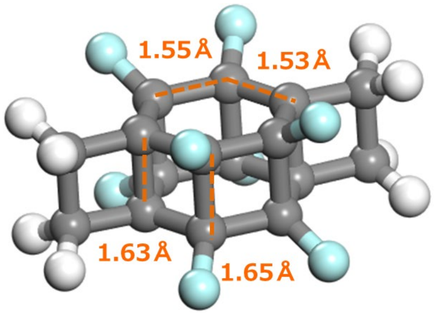

Figure S4. C···C distances in **11** optimised by MOPAC PM7.

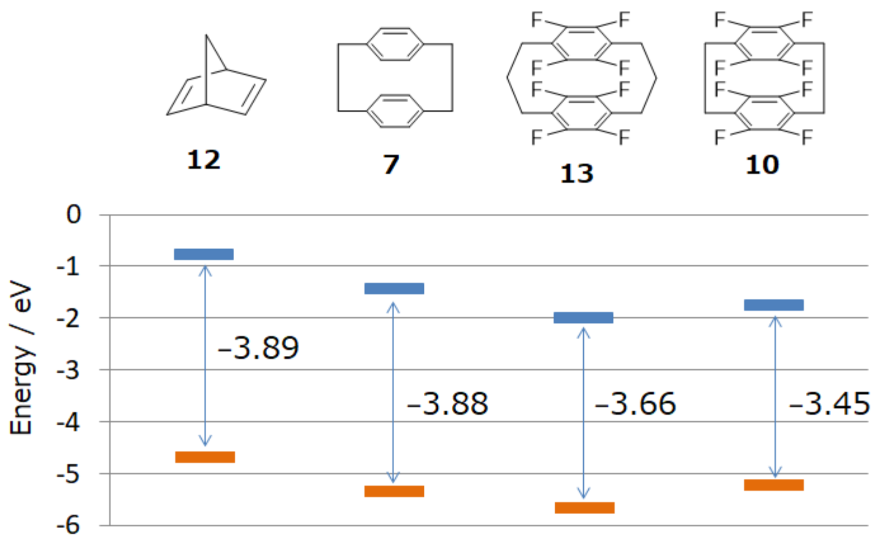

Figure S5. Highest occupied molecular orbital (HOMO)–LUMO gaps of selected cyclophanes.

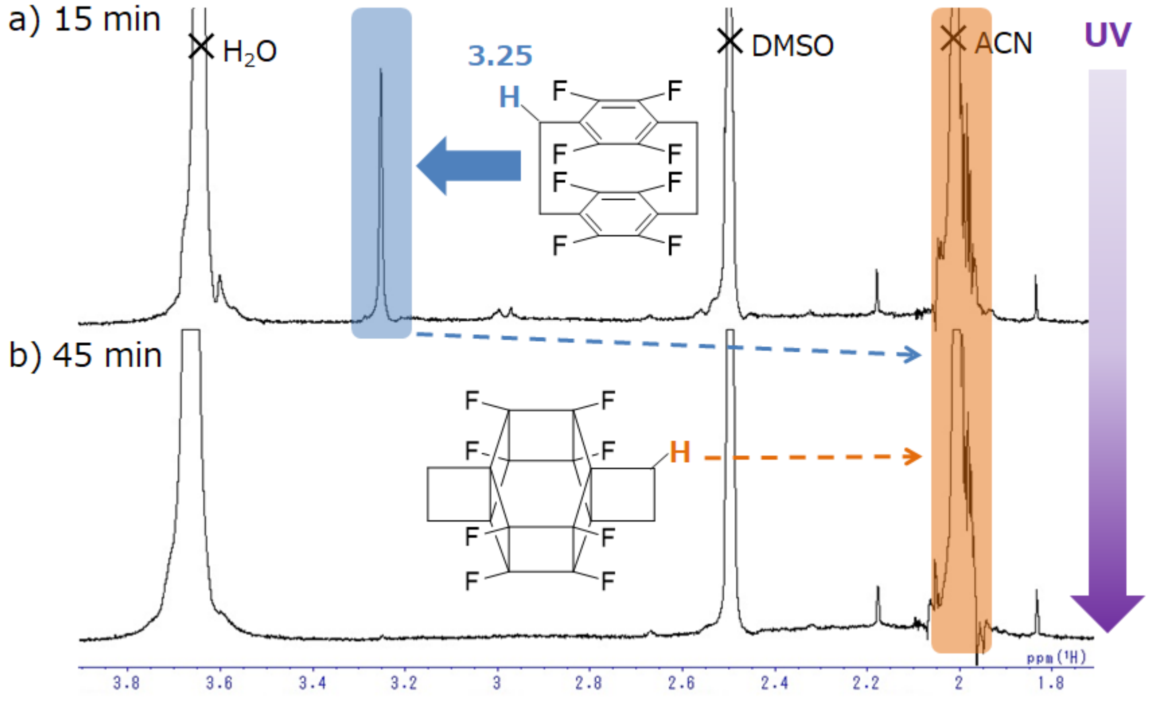

Figure S6. ¹H NMR spectra (400 MHz, $CD_3CN/D_2O/(CD_3)_2SO$ = 2/1/8, v/v/v) of **10** recorded after (**a**) 15 and (**b**) 45 min irradiation.

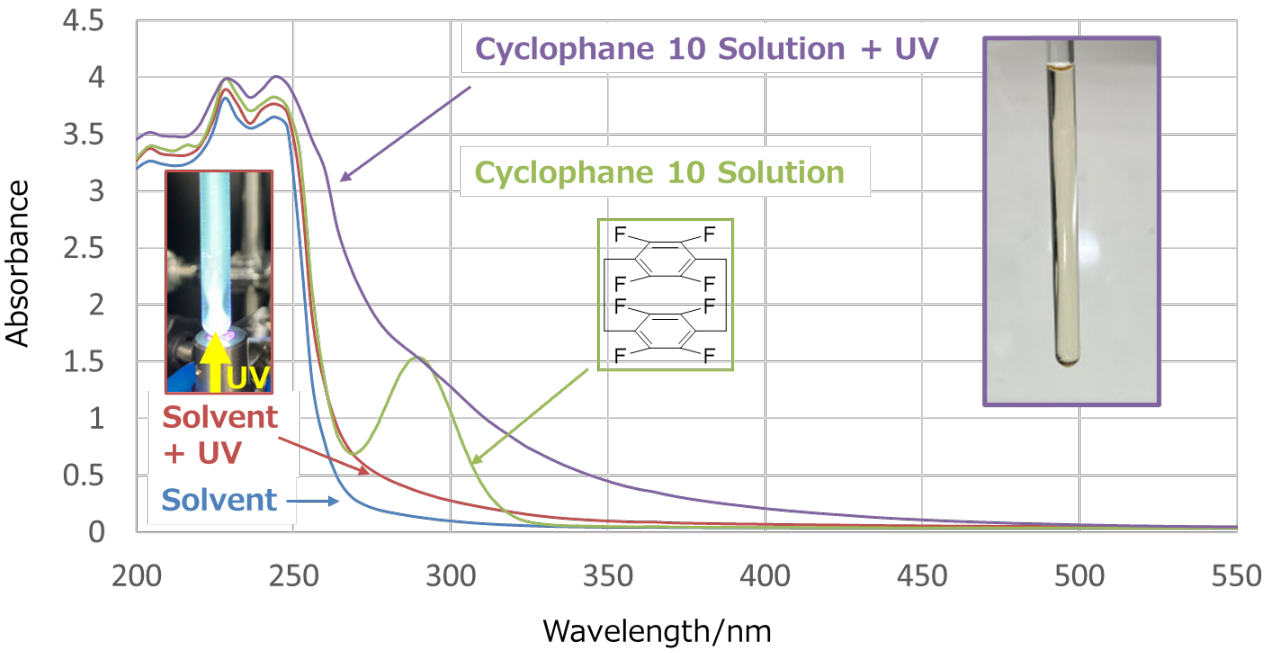

Figure S7. Ultraviolet (UV)–Vis spectra of selected reaction systems.

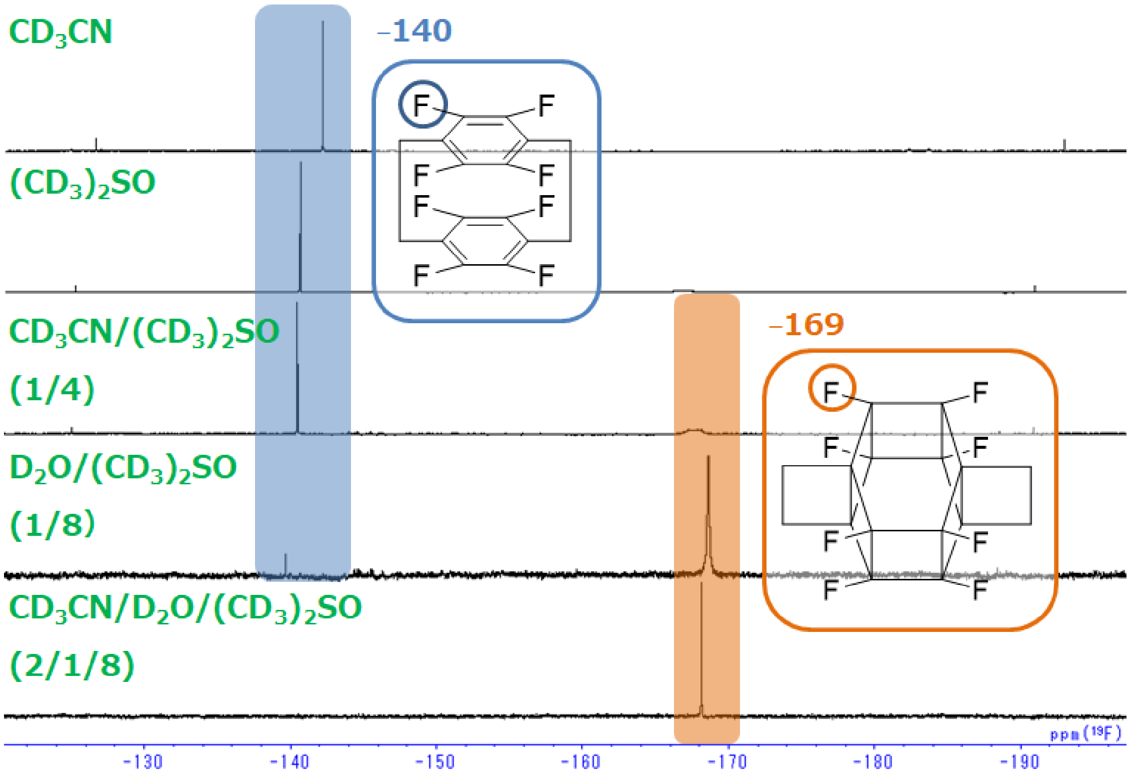

Figure S8. ^{19}F NMR spectra (373 MHz) of **10** photoirradiated in different solvents.

(a) $CD_3CN/H_2O/DMSO$-d6

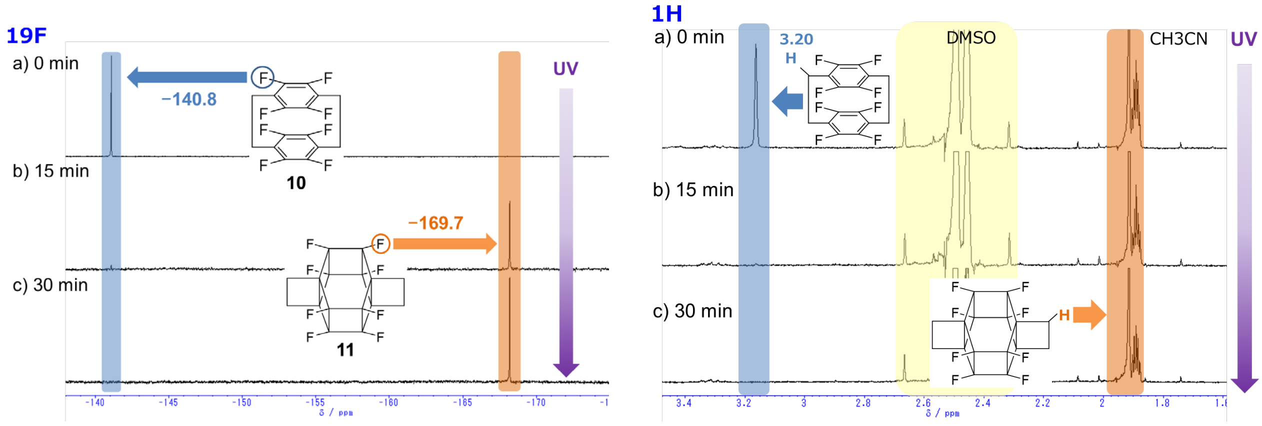

(b) $D_2O/DMSO$-d6

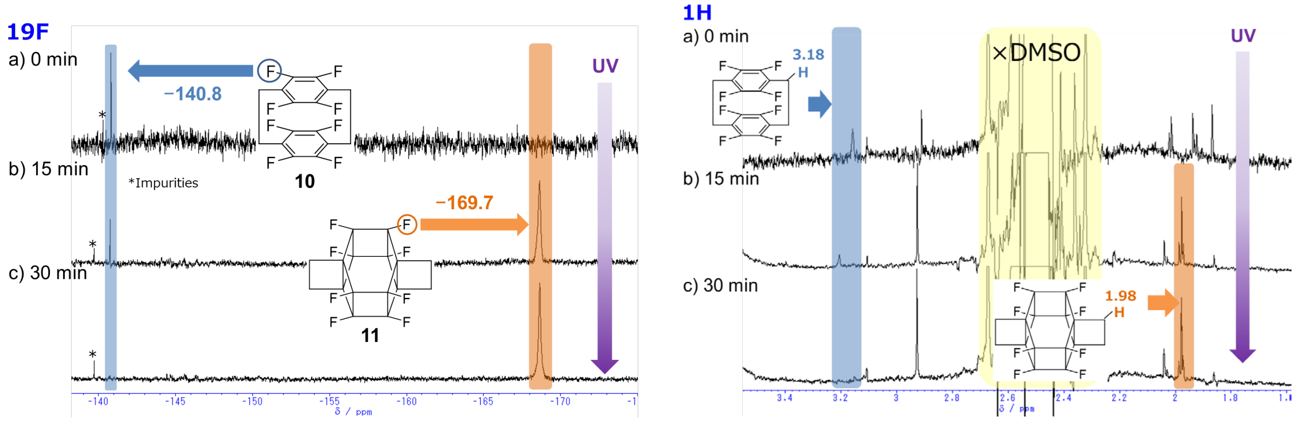

Figure S9. NMR spectra (400 MHz) of **10** in (**a**) $CD_3CN/D_2O/DMSO$-d_6 and (**b**) $D_2O/DMSO$-d_6.

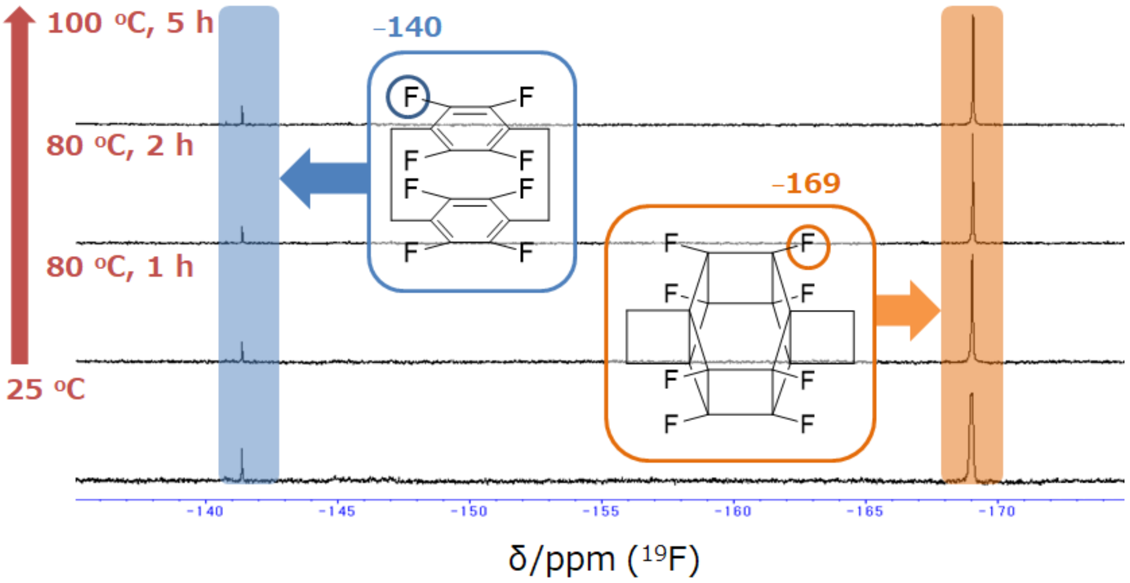

Figure S10. ^{19}F NMR spectra (373 MHz) of the photoreaction mixture recorded before and after heating.

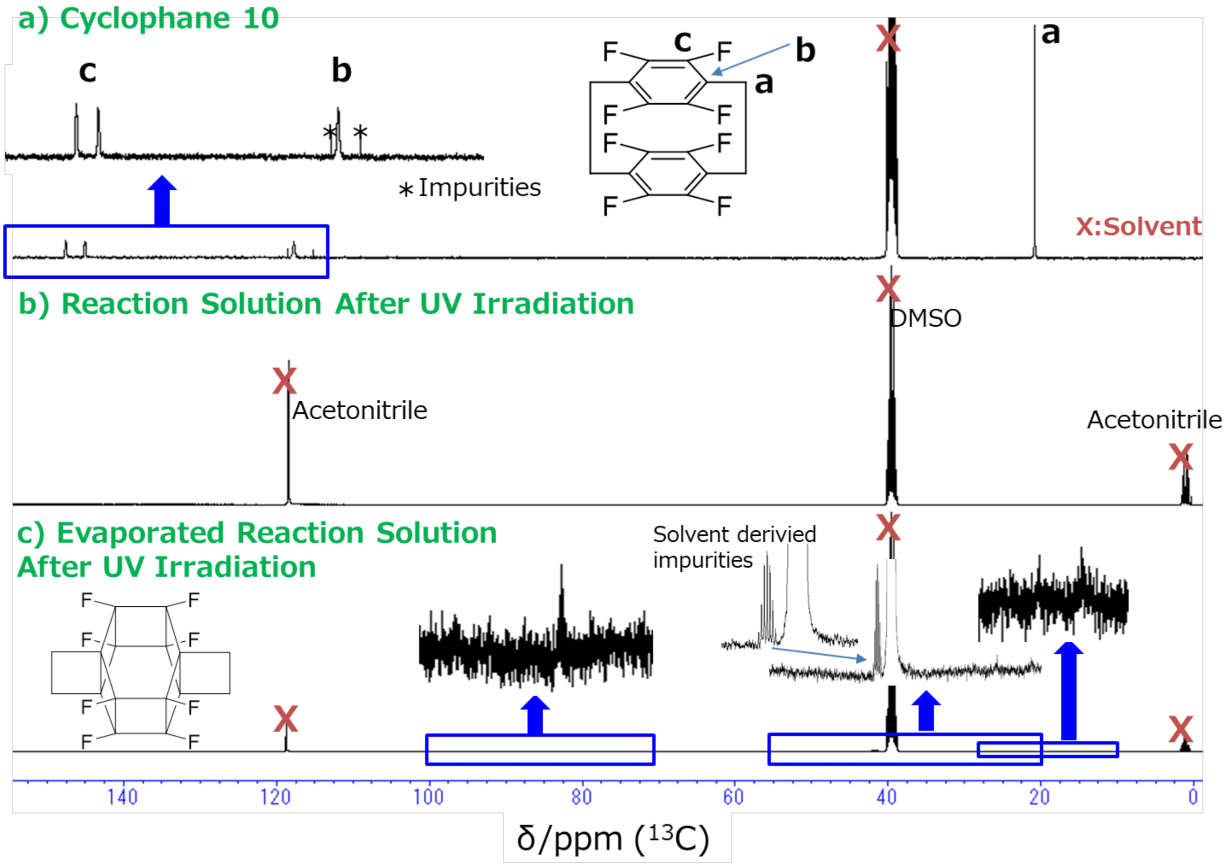

Figure S11. ^{13}C NMR spectra (100 MHz) of (**a**) **10**, (**b**) reaction solution after UV irradiation, and (**c**) evaporated reaction solution.

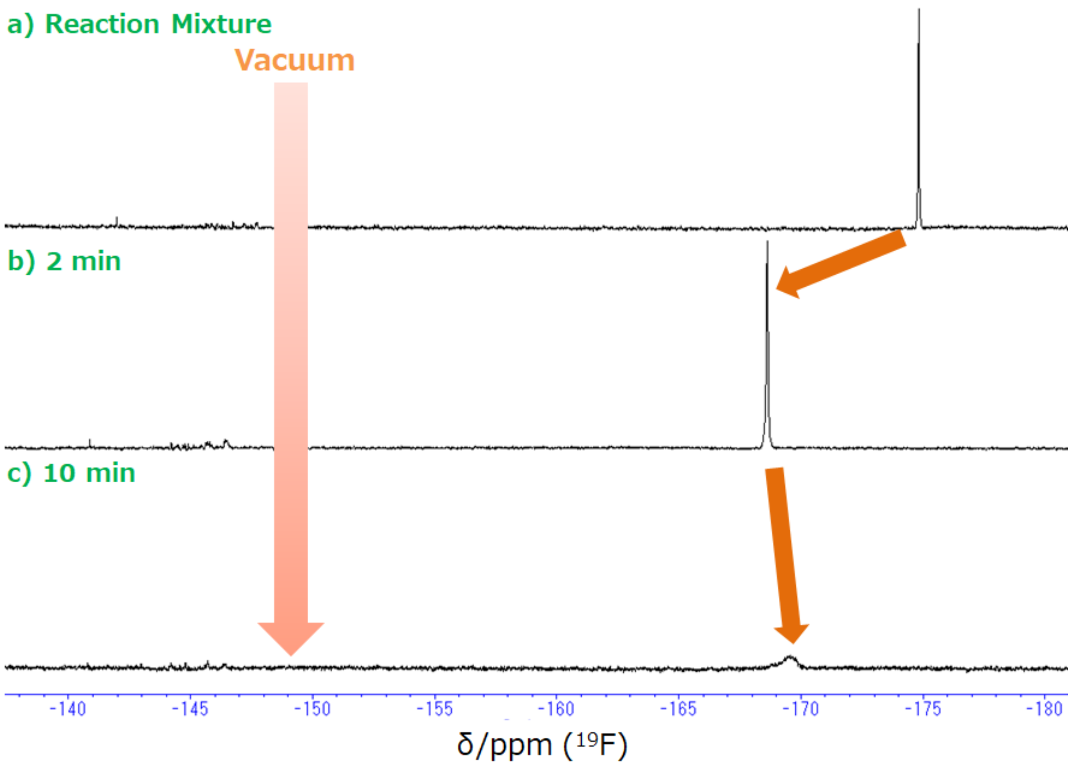

Figure S12. ^{19}F NMR spectra (373 MHz) of the reaction solution kept under vacuum for (**a**) 0, (**b**) 2, and (**c**) 10 min.

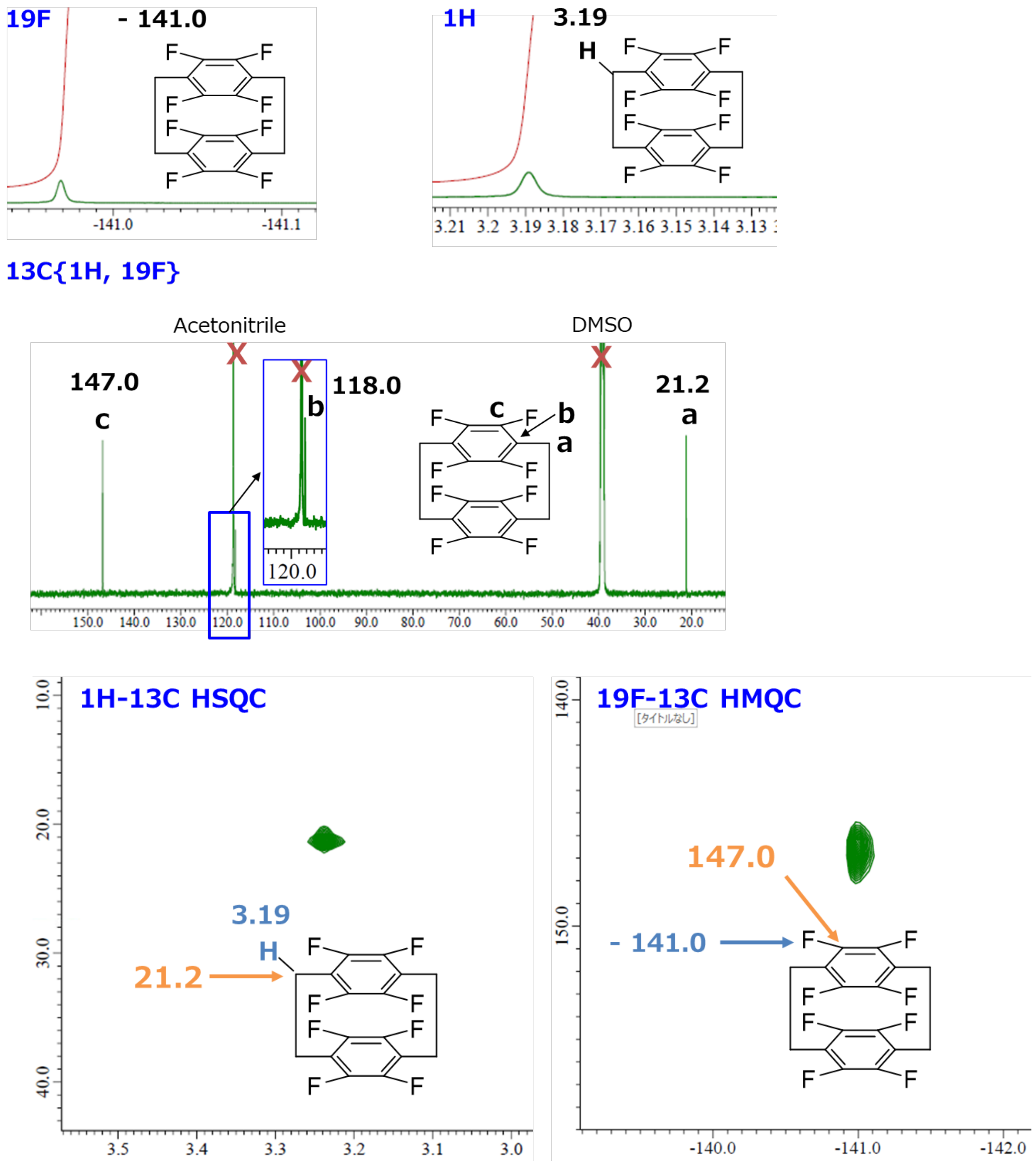

Figure S13. NMR spectra of cyclophane **10** assigned by ROYAL probe HFX at 25 °C. Data points for 1H: 16 k, 19F: 32 k, 13C{1H, 19F}: 32 k, 1H-13C HSQC: 1024 x 128, 19F-13C HMQC: 1024 x 64. Scans for 1H and 19F: 8, 13C{1H, 19F}: 11 k, 1H-13C HSQC: 4, 19F-13C HMQC: 4.

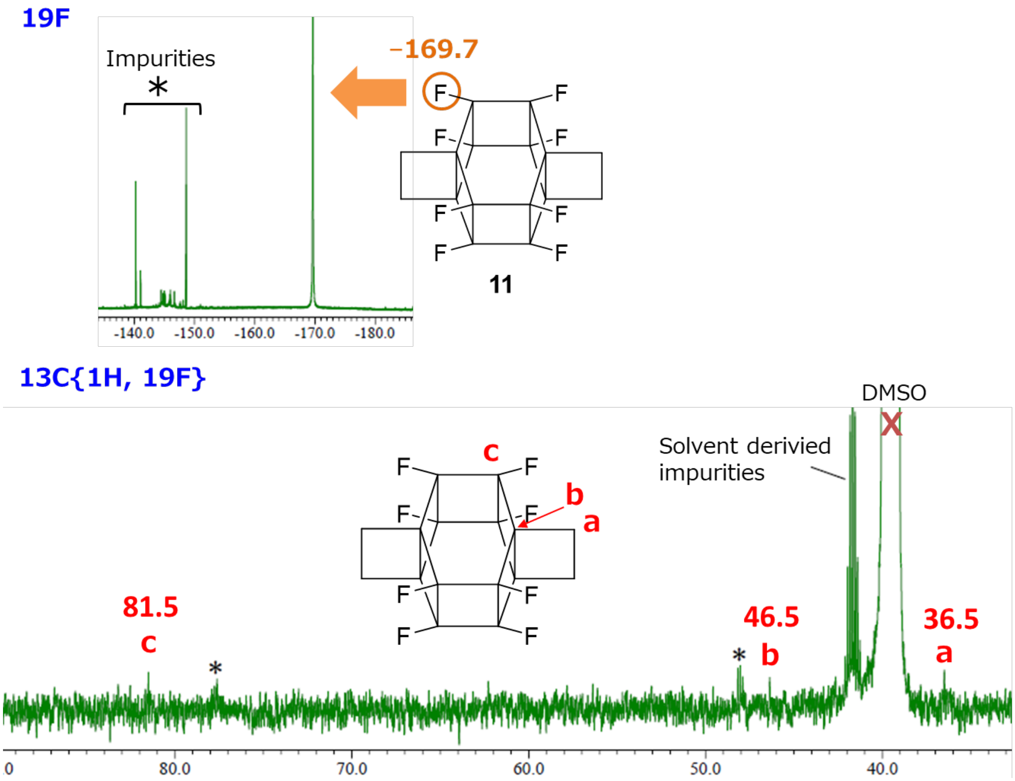

Figure S14. NMR spectra of **11** by ROYAL probe HFX at 25 °C. Data points for 1H{19F}: 20,480, 19F{1H}: 65,536, 13C{1H, 19F}: 47,162.Scans for 1H{19F}: 1024, 19F{1H}: 1024, 13C{1H, 19F}: 65,536.

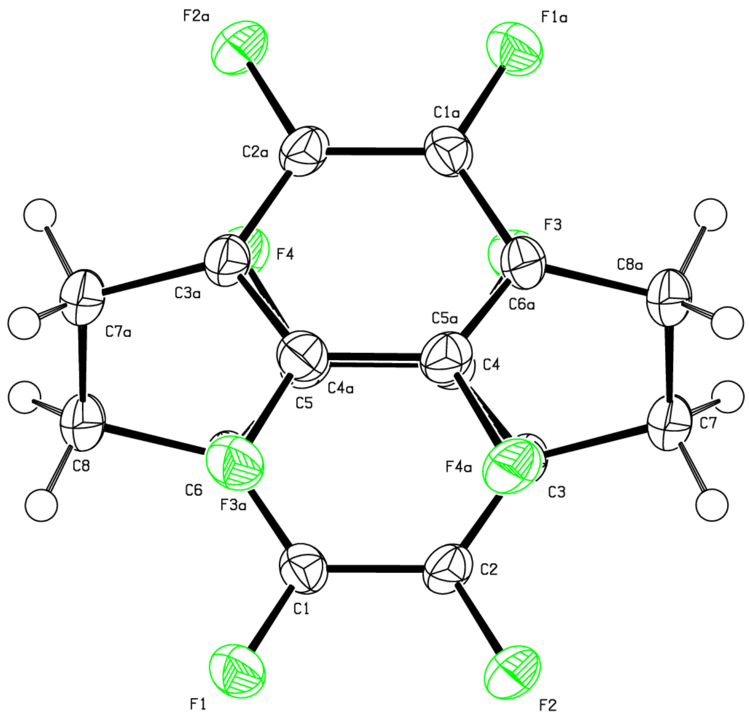

Figure S15. ORTEP drawing of **10** (50% probability).

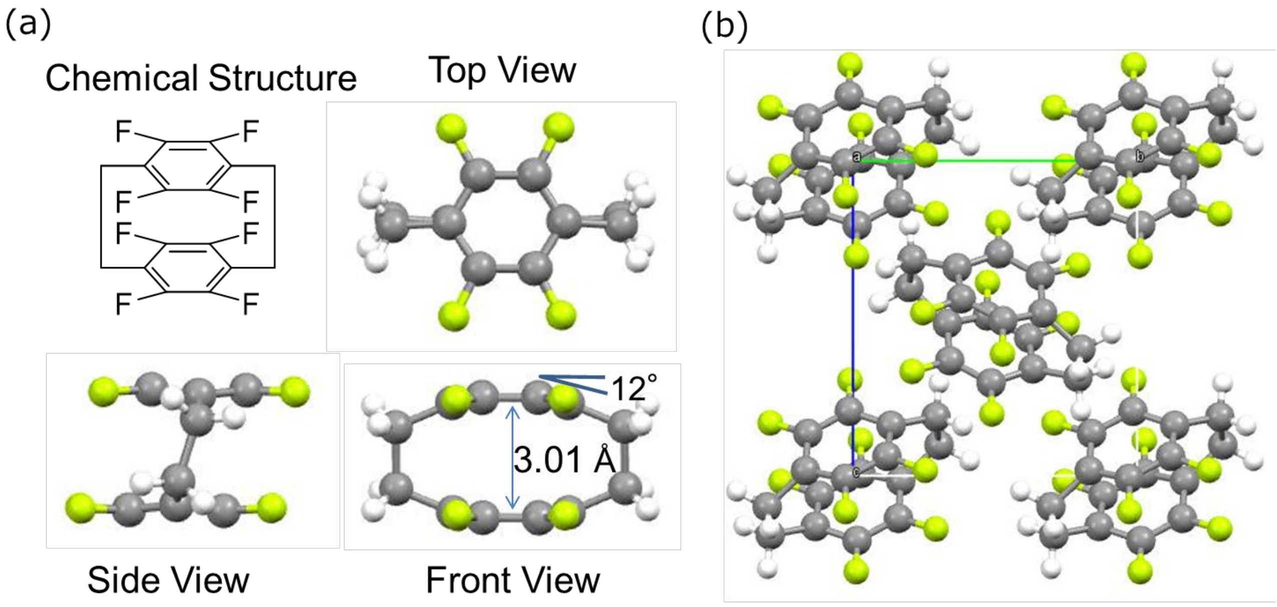

Figure S16. (**a**) Single-molecule and (**b**) packing (*a*-axis) structures of **10**. Colourless, platelets, $C_{16}H_8F_8$, $M = 352.23$, monoclinic, $P2_1/n$ (#14), $a = 8.5348(10)$ Å, $b = 8.1715(11)$ Å, $c = 9.1344(13)$ Å, $\beta = 96.829(4)$°, $V = 632.54(14)$ Å^3, $Z = 2$, $D_{calc} = 1.849$ g/cm^3, $\mu = 1.898$ cm^{-1}, R $(wR) = 0.0811$ (0.1994) for 1440 reflections. CCDC Deposition Number: 2003703.

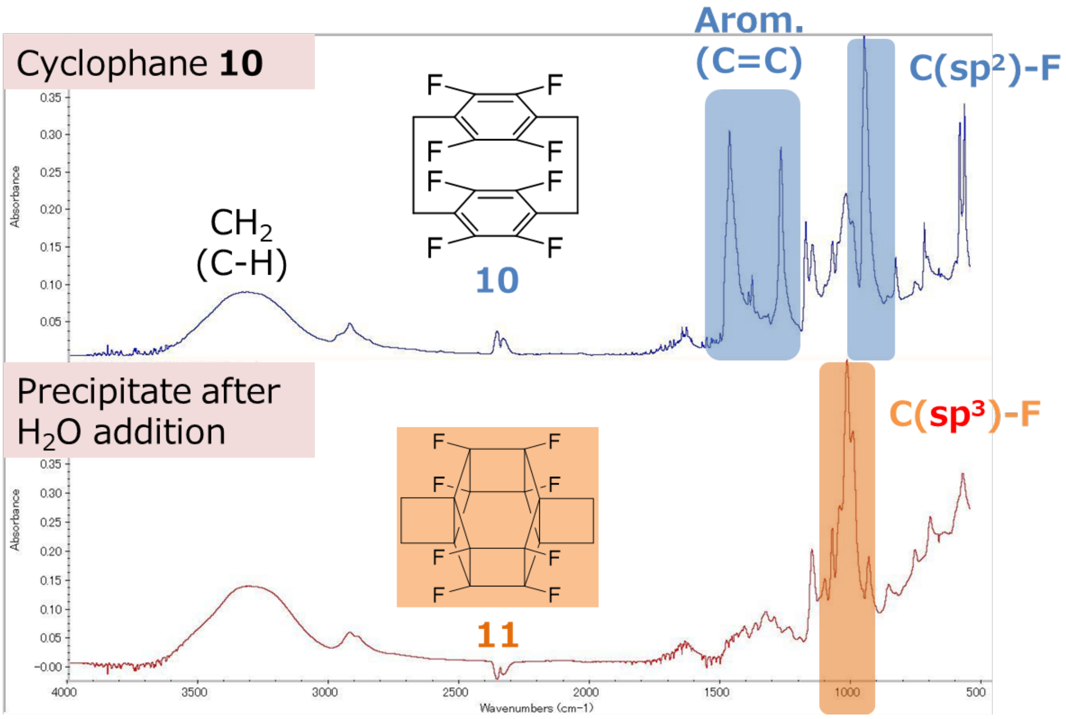

Figure S17. Infrared spectra of **10** and the recovered precipitate.

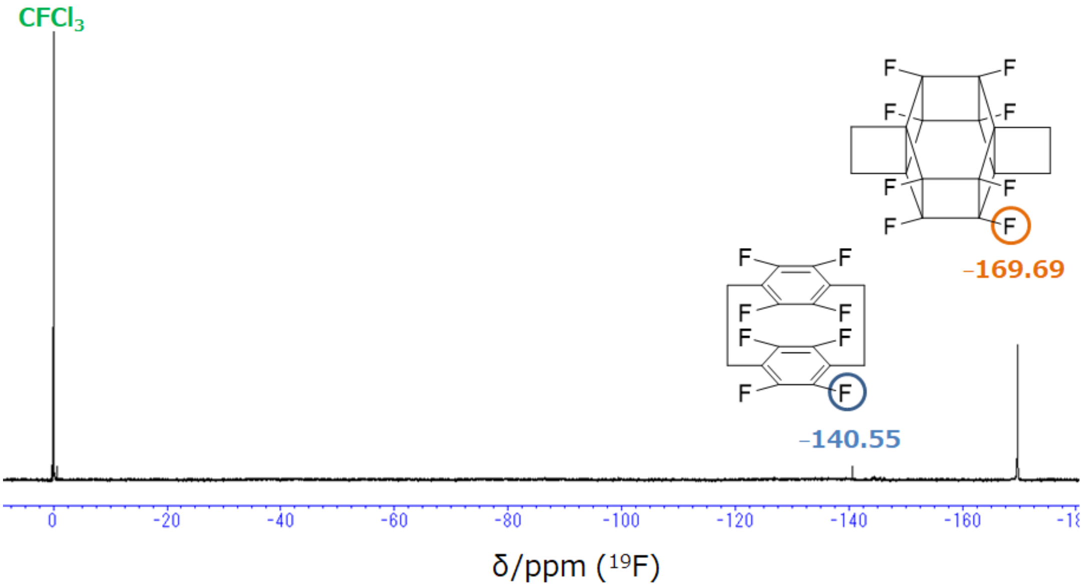

Figure S18. ^{19}F NMR (373 MHz) spectrum of the photoreaction solution with a $CFCl_3$ standard.

Table S1. Cartesian coordinates of the PM7 optimized geometry of norbornadiene **12** (in Å).

NO.	ATOM	X	Y	Z
1	C	-7.101317826	0.928463820	0.175665950
2	C	-6.710551195	-0.135217724	-0.859277263
3	C	-5.317990056	0.290631548	-1.343653094
4	C	-4.471287619	0.179464601	-0.303137916
5	C	-6.254568531	0.817282463	1.216142083
6	C	-5.277184219	-0.323470085	0.902182242
7	C	-6.272951256	-1.282707104	0.136028837
8	H	-7.909792764	1.603455599	0.010354579
9	H	-7.445543553	-0.383937383	-1.617551460
10	H	-5.145745295	0.614969570	-2.344555315
11	H	-3.428365322	0.389737572	-0.234030718
12	H	-6.192317460	1.378195906	2.120717632
13	H	-4.719019995	-0.742084413	1.733060189
14	H	-5.784274138	-2.127703803	-0.351879033
15	H	-7.086166444	-1.662097084	0.757053834

Table S2. Cartesian coordinates of the PM7 optimized geometry of quadricyclane **13** (in Å).

NO.	ATOM	X	Y	Z
1	C	-5.560738385	1.483308628	-1.764834230
2	C	-5.262503158	2.198369600	-0.443988144
3	C	-5.293119332	0.667885506	-0.481961980
4	C	-6.795832071	0.294953635	-0.405562659
5	C	-7.063435872	1.110346226	-1.688400683
6	C	-7.533005529	1.634941243	-0.328507945
7	C	-6.568403045	2.715635570	0.157226597
8	H	-5.016063493	1.563434976	-2.673340858
9	H	-4.356044990	2.756805594	-0.278123513
10	H	-4.504701202	0.005088128	-0.221958651
11	H	-7.155211675	-0.652645968	-0.087163393
12	H	-7.666676277	0.905578394	-2.538462033
13	H	-8.574767986	1.709872166	-0.063697267
14	H	-6.527088560	2.774584248	1.255818587
15	H	-6.835507151	3.713988180	-0.222172614

Table S3. Cartesian coordinates of the PM7 optimized geometry of fluorinated norbornadiene **14** (in Å).

NO.	ATOM	X	Y	Z
1	C	-7.065374054	1.052923231	0.157011271
2	C	-6.684782851	-0.000888288	-0.885623041
3	C	-5.289613135	0.417949005	-1.355441633
4	C	-4.440384648	0.305936206	-0.311877360
5	C	-6.216089965	0.941543573	1.200562738
6	C	-5.232338188	-0.191718598	0.899572986
7	C	-6.236115686	-1.143498960	0.120905665
8	F	-8.054779586	1.884766135	-0.021997656
9	H	-7.423267464	-0.254199771	-1.646353099
10	F	-5.055256495	0.813523419	-2.576734249
11	F	-3.162483717	0.563449343	-0.251279754
12	F	-6.161643562	1.637443957	2.303190253
13	H	-4.673307201	-0.615234840	1.734130818
14	H	-5.750007548	-1.994139289	-0.365620270
15	H	-7.050053634	-1.528963306	0.741863057

Table S4. Cartesian coordinates of the PM7 optimized geometry of fluorinated quadricyclane **15** (in Å).

NO.	ATOM	X	Y	Z
1	C	-5.394730613	1.472595175	-1.810959559
2	C	-5.195649683	2.187674664	-0.465259721
3	C	-5.126563397	0.653315653	-0.519249007
4	C	-6.602455424	0.177185779	-0.514975420
5	C	-6.870492701	0.996464311	-1.806695794
6	C	-7.442783189	1.462727808	-0.458737441
7	C	-6.565866241	2.594652339	0.077417965
8	F	-4.677816036	1.640316555	-2.888555047
9	H	-4.337876288	2.809090732	-0.249481499
10	F	-4.112215538	-0.087531231	-0.164821880
11	F	-6.990898858	-1.016299365	-0.157148566
12	F	-7.556282778	0.711488556	-2.879869734
13	H	-8.500850568	1.466062256	-0.237573989
14	H	-6.578478642	2.644579721	1.179942990
15	H	-6.885576097	3.582200997	-0.297662850

Table S5. Cartesian coordinates of the PM7 optimized geometry of cyclophane **10** (in Å).

NO.	ATOM	X	Y	Z
1	C	-5.269857471	0.637797419	-0.675458665
2	C	-5.219822419	-0.758013599	-0.774322948
3	C	-4.704890812	-1.543777147	0.264564962
4	C	-4.562429891	-0.895354684	1.497990735
5	C	-4.612420593	0.500459529	1.596918561
6	C	-4.806911929	1.304117961	0.466302058
7	F	-4.352301896	1.053467321	2.765727627
8	F	-4.257777253	-1.587782703	2.578381259
9	F	-5.653570693	1.325272401	-1.733590079
10	F	-5.558962820	-1.315273848	-1.920567295
11	C	-1.744267339	0.663780120	0.772206363
12	C	-1.693758444	-0.732178580	0.675771760
13	C	-2.156261416	-1.400622858	-0.464890875
14	C	-2.350804373	-0.599045935	-1.596914192
15	C	-2.401578849	0.796923584	-1.500432067
16	C	-2.259578114	1.447450581	-0.268114561
17	F	-2.706210800	1.486720142	-2.582501552
18	F	-2.610064350	-1.154094034	-2.764864921
19	F	-1.404865437	1.223576880	1.917205499
20	F	-1.309319318	-1.418088928	1.734666054
21	C	-4.134773927	-2.899976826	0.017573288
22	C	-4.336181785	2.718762140	0.415141263
23	C	-2.830380611	2.803784908	-0.023221287
24	C	-2.626401460	-2.815392156	-0.412074351
25	H	-4.216261210	-3.546021363	0.915994203
26	H	-4.701270148	-3.443230093	-0.766704858
27	H	-4.948066121	3.326376093	-0.283161354
28	H	-4.453100618	3.222798744	1.396714234
29	H	-2.260198509	3.351008188	0.755712075
30	H	-2.754947087	3.446209663	-0.924785878
31	H	-2.018405677	-3.420759859	0.291554948
32	H	-2.503413699	-3.322208214	-1.391462096

Table S6. Cartesian coordinates of the PM7 optimized geometry of prismane **11** (in Å).

NO.	ATOM	X	Y	Z
1	C	-0.412676373	1.281677479	-0.373296389
2	C	-2.062293736	0.373096988	-1.624013025
3	C	-3.061168323	-0.331246656	-0.710549391
4	C	-0.554659778	0.067170124	-1.481955773
5	C	-1.920359798	1.587626498	-0.515364567
6	C	-2.920881492	0.869354481	0.385457076
7	C	-2.453467164	-0.132664756	1.437209128
8	C	-1.087796612	-1.653064176	0.470638984
9	C	-0.087320197	-0.934809450	-0.430266868
10	C	-2.595434028	-1.347117473	0.328460602
11	C	-0.945826946	-0.438579150	1.579364438
12	C	0.053070239	0.265798110	0.665755661
13	F	0.125865584	0.114938925	-2.610097558
14	F	0.382488648	2.306344399	-0.609642566
15	F	-2.457159622	0.639097303	-2.853385460
16	F	-0.550721305	-0.704959033	2.808590001
17	F	-0.807106448	-2.896261308	0.807947373
18	F	-3.134755694	-0.180677318	2.564854784
19	F	-3.390498081	-2.371887115	0.564510306
20	F	-2.201365253	2.830552930	-0.853204581
21	C	1.544837174	-0.058539300	0.787474179
22	C	1.409352828	-1.217104537	-0.270360794
23	C	-4.552931293	-0.007148842	-0.832351786
24	C	-4.417608010	1.151508756	0.225505043
25	H	1.866102333	-0.392996186	1.779902808
26	H	2.220114230	0.748899887	0.484003467
27	H	1.653582905	-2.211228606	0.119597009
28	H	2.007252107	-1.069271027	-1.176290764
29	H	-4.874090674	0.327207899	-1.824854852
30	H	-5.228134161	-0.814617565	-0.529042279
31	H	-5.015784829	1.003659674	1.131200405
32	H	-4.661930336	2.145478784	-0.164706984

Table S7. Cartesian coordinates of the PM7 optimized geometry of cyclophane **7** (in Å).

NO.	ATOM	X	Y	Z
1	C	-5.269703583	0.637811050	-0.675506783
2	C	-5.219961380	-0.750389220	-0.773827474
3	C	-4.697330636	-1.500217507	0.285343831
4	C	-4.551942000	-0.890128599	1.535916088
5	C	-4.601706588	0.498122857	1.634312448
6	C	-4.796454108	1.266232986	0.481372037
7	H	-4.402487318	0.983289044	2.585867823
8	H	-4.313872608	-1.489143676	2.410532038
9	H	-5.591703536	1.231936653	-1.526225559
10	H	-5.503191458	-1.240219397	-1.701331419
11	C	-1.728821121	0.661411142	0.811071518
12	C	-1.678640668	-0.726960820	0.714824969
13	C	-2.149567857	-1.357180157	-0.441937524
14	C	-2.342383525	-0.590860036	-1.596391473
15	C	-2.392825760	0.797465690	-1.500092905
16	C	-2.249818536	1.409419185	-0.250137427
17	H	-2.629177498	1.394955066	-2.376209727
18	H	-2.539236692	-1.077467562	-2.547614496
19	H	-1.447521036	1.152782624	1.738392672
20	H	-1.358162728	-1.319927340	1.566973797
21	C	-4.127066115	-2.863676756	0.037816001
22	C	-4.326026279	2.688125038	0.431256496
23	C	-2.821033926	2.773061679	-0.005641852
24	C	-2.619627274	-2.779163034	-0.390786201
25	H	-4.208763894	-3.496061873	0.941851306
26	H	-4.695638102	-3.392177514	-0.750390465
27	H	-4.938739989	3.281997035	-0.272859161
28	H	-4.442329381	3.177396358	1.416700388
29	H	-2.249060102	3.306275507	0.777001627
30	H	-2.745294669	3.401143613	-0.913236399
31	H	-2.010655747	-3.371119031	0.318189328
32	H	-2.497632943	-3.270813232	-1.374313273

Table S8. Cartesian coordinates of the PM7 optimized geometry of cyclophane **16** (in Å).

NO.	ATOM	X	Y	Z
1	C	-6.640698517	-0.372018230	-0.472126500
2	C	-5.905883962	-1.554511159	-0.617661820
3	C	-5.008400146	-1.995522732	0.361115563
4	C	-5.045016938	-1.291512823	1.569238943
5	C	-5.819150631	-0.136778404	1.732951814
6	C	-6.562305100	0.413297786	0.682701879
7	F	-5.806563126	0.446983538	2.916637067
8	F	-4.316634576	-1.689241970	2.595946764
9	F	-7.401912609	0.002059172	-1.483751074
10	F	-6.062077276	-2.239682284	-1.734818646
11	C	-4.220169067	1.341756648	-1.592757057
12	C	-3.446524467	0.186697433	-1.756692746
13	C	-2.703320020	-0.363798455	-0.706697855
14	C	-2.624581155	0.421433919	0.448241625
15	C	-3.359642798	1.603730551	0.594300466
16	C	-4.257002044	2.045188197	-0.384344780
17	F	-3.203512509	2.288636562	1.711715268
18	F	-1.863173734	0.046788106	1.459558018
19	F	-4.948367521	1.739565057	-2.619518637
20	F	-3.459406169	-0.396669990	-2.940516411
21	C	-4.055504888	-3.117251044	0.118358327
22	C	-7.198416226	1.759252091	0.778000261
23	C	-5.210125948	3.166784834	-0.141427413
24	C	-2.067700637	-1.709999867	-0.802628800
25	C	-3.077096882	-2.849730766	-1.042057609
26	C	-6.189293640	2.899139538	1.018242514
27	H	-4.627689190	-4.047576860	-0.104243077
28	H	-3.468081864	-3.363182901	1.028133911
29	H	-7.941763457	1.759368279	1.608643868
30	H	-7.794159531	1.998349547	-0.128171008
31	H	-4.637957303	4.097032118	0.081826789
32	H	-5.796979946	3.413024260	-1.051502585
33	H	-1.325349152	-1.710188923	-1.634171937
34	H	-1.471011378	-1.949239656	0.102950241
35	H	-3.630064134	-2.684156959	-1.982754592

36	H	-2.500584842	-3.782539856	-1.222799434
37	H	-5.636788841	2.733603127	1.959264711
38	H	-6.766217221	3.831755528	1.198644926
